# Analysis of subjects with menstrually related migraine vs. Non-menstrually related migraine treated with MAP0004

**DOI:** 10.1186/1129-2377-14-S1-P143

**Published:** 2013-02-21

**Authors:** S Aurora, B Lu, E Connors, X Li, D Kellerman, S Kori

**Affiliations:** 1 Swedish Neuroscience Institute, Swedish Headache Center, Seattle, WA, USA; 2 MAP Pharmaceuticals, USA

## 

Menstrually related migraine (MRM) is defined as occurring from days -2 to +3 of menstruation in at least 2 out of 3 menstrual cycles, and additionally at other times of the menstrual cycle. MRM is generally longer lasting, more severe, and more difficult to treat compared to non-MRM attacks. MAP0004 is an investigational orally inhaled dihydroergotamine (DHE) for the acute treatment of migraine. In a large Phase 3 study, MAP0004 was effective and well tolerated in treating an acute migraine attack compared to placebo. This post-hoc analysis compares the efficacy of MAP0004 in treating MRM versus non-MRM, including an analysis of recurrence rates using 4 different, previously published recurrence rate definitions. The efficacy of MAP0004, as measured by pain relief at 2 hours, pain free at 2 hours, sustained pain relief at 2-24 and 2-48 hours, and sustained pain free at 2-24 and 2-48 hours values, was not significantly different between subjects with MRM and non-MRM. Furthermore, the MRM recurrence rates after pain relief were not statistically higher than that of non-MRM treated with MAP0004. There were no significant differences in the frequency of adverse events for MRM vs. non-MRM subjects, and no drug-related serious adverse events were reported. In the study, MAP0004 was effective and well-tolerated for both MRM and non-MRM.

## References

[B1] Headache Classification Subcommittee of the International Headache Society The international classification of headache disorders Cephalalgia 2004 14 suppl 1

[B2] Granella F Characteristics of menstrual and nonmenstrual attacks in women with menstrually related migraine referred to headache centres Cephalalgia 2004 14 9 707 716 10.1111/j.1468-2982.2004.00741.x 15315526

[B3] Sacco S Migraine in women: the role of hormones and their impact on vascular diseases J Headache Pain 2012 14 3 177 89 Epub2012Feb26 10.1007/s10194-012-0424-y 22367631 PMC3311830

